# Postoperative adjuvant therapy for resectable early non-small cell lung cancer: A protocol for a systematic review and meta-analysis

**DOI:** 10.1097/MD.0000000000016468

**Published:** 2019-07-26

**Authors:** Tianci Chai, Peipei Zhang, Yuhan Lin, Zhenyang Zhang, Wenwei Lin, Mingqiang Kang, Jiangbo Lin

**Affiliations:** a Department of Thoracic Surgery, Fujian Medical University Union Hospital; b The Graduate School of Fujian Medical University; c School of Stomatology, Fujian Medical University, Fuzhou, China.

**Keywords:** chemotherapy, immunotherapy, molecular targeted therapy, non-small cell lung cancer, radiotherapy

## Abstract

**Background:**

Lung cancer is one of the most common malignant tumors, and non-small cell lung cancer (NSCLC) accounts for about 85% of lung cancer diagnosed. For patients with resectable early stage non-small cell lung cancer, routine postoperative adjuvant therapy can significantly prolong overall patient survival and reduce the risk of cancer recurrence. With the emergence and maturity of molecular targeted therapy and immunotherapy, the postoperative chemotherapy strategy of lung cancer patients has changed a lot. To evaluate the efficacy of postoperative adjuvant therapy (platinum-based chemotherapy, platinum-based chemotherapy plus molecular targeted therapy, platinum-based chemotherapy plus anti-angiogenic agents, or platinum-based chemotherapy plus immunotherapy) with or without radiotherapy for patients with NSCLC, we will conduct a systematic review and meta-analysis of the published or unpublished relevant randomized controlled trials.

**Methods:**

We will search PubMed (Medline), Embase, Google Scholar, Cancerlit, and the Cochrane Central Register of Controlled Trials for related studies published without language restrictions before June 20, 2019. Two review authors will search and assess relevant studies independently. Randomized controlled trials (RCTs) and quasi-RCTs studies will be included. We will perform subgroup analysis in different methods of postoperative adjuvant therapy for patients with resectable early NSCLC. Because this study will be based on published or unpublished records and studies, there is no need for ethics approval.

**Results:**

The results of this study will be published in a peer-reviewed journal.

**Conclusion:**

This study will comprehensively compare the efficacy of platinum-based chemotherapy with that of molecular targeted therapy and immunotherapy for patients after surgery with resectable early NSCLC. Since large-sample randomized trials meeting the inclusion criteria of this study may be insufficient, we will consider incorporating some high-quality small-sample-related trials, which may lead to high heterogeneity and affect the reliability of the results.

## 1 Introduction

Worldwide, lung cancer is the second most common malignancy and the leading cause of cancer-related death.^[1]^ Non-small cell lung cancer (NSCLC) is the most common pathological type of lung cancer,^[2]^ accounting for about 85% of new cases of lung cancer diagnosed every year.^[3]^

Surgical resection of early resectable NSCLC provides potential therapeutic opportunities for patients considered to be the optimum treatment.^[4]^ The 5-year survival rate of patients with pathological stage Ia to IIIa NSCLC after surgery is 73% to 25%.^[5,6]^ However, there is no appropriate cure for postoperative recurrence or metastasis of NSCLC.

With postoperative adjuvant platinum-based chemotherapy which has been used as a routine postoperative adjuvant therapy after surgery, the 5-year survival rate of patients can be improved by roughly 5%.^[7,8]^

However, adjuvant radiotherapy is not recommended for postoperative adjuvant therapy for early non-small cell lung cancer because it can significantly increase the 2-year mortality of patients by about 7%.^[6,9,10]^ With the emergence of molecular targeted therapy and immunotherapy, the postoperative adjuvant therapy has become diversified.^[11]^ At present, although there is no sufficient evidence support EGFR-TKIs, immunomodulation, and angiogenesis inhibitors can effectively improve the outcome following surgery, additional high quality trials are ongoing or have been published.

To evaluate the efficacy of postoperative adjuvant therapy (platinum-based chemotherapy, platinum-based chemotherapy plus molecular targeted therapy, platinum-based chemotherapy plus anti-angiogenic agents, or platinum-based chemotherapy plus immunotherapy) with or without radiotherapy for patients with NSCLC and obtain reliable evidence, we will conduct a systematic review and meta-analysis of published or unpublished related trials, and then integrate these postoperative treatment methods for patients with NSCLC, so as to provide a reference for clinicians to formulate the optimum treatment strategy.

## 2 Objective

We will evaluate the efficacy of postoperative adjuvant therapy (platinum-based chemotherapy, platinum-based chemotherapy plus molecular targeted therapy, platinum-based chemotherapy plus anti-angiogenic agents, or platinum-based chemotherapy plus immunotherapy) with or without radiotherapy for patients with NSCLC.

## 3 Methods

This protocol is conducted according to the Preferred Reporting Items for Systematic Review and Meta-Analysis Protocols (PRISMA-P) statement.^[12]^ We will report the results of this systematic review and meta-analysis adhere to the Preferred Reporting Items for Systematic Reviews and Meta-Analyse (PRISMA) guidelines.^[13]^ This protocol has been registered in the PROSPERO network (registration number: CRD42018118121).

### 3.1 Patient and public involvement

This study will be based on published or unpublished studies and records and will not involve patients or the public directly.

### 3.2 Eligibility criteria

#### 3.2.1 Types of studies

Randomized controlled trials (RCTs) and quasi-RCTs published or unpublished will be included, which have been completed and compared postoperative platinum-base chemotherapy versus platinum-based chemotherapy plus molecular targeted therapy, platinum-based chemotherapy versus platinum-based chemotherapy plus anti-angiogenic agents, or platinum-based chemotherapy versus platinum-based chemotherapy plus immunotherapy for patients with NSCLC.

#### 3.2.2 Types of participants

The participants will be adults diagnosed with resectable early NSCLC histologically or cytologically confirmed who were treated with platinum-based chemotherapy, platinum-based chemotherapy plus molecular targeted therapy, platinum-based chemotherapy plus anti-angiogenic agents, or platinum-based chemotherapy plus immunotherapy after surgery. No restrictions on ethnicity, sex, education, and economic status will be applied.

#### 3.2.3 Types of interventions

According to the means of postoperative adjuvant therapy for patients with resectable early NSCLC, the trials included will be divided into the following categories.

- postoperative platinum-base chemotherapy versus platinum-based chemotherapy plus molecular targeted therapy
- platinum-based chemotherapy versus platinum-based chemotherapy plus anti-angiogenic agents
- platinum-based chemotherapy versus platinum-based chemotherapy plus immunotherapy.

#### 3.2.4 Types of outcome measures

##### 3.2.4.1 Primary outcomes

The primary outcomes will be postoperative overall survival of patients with resectable early NSCLC who were treated with adjuvant therapy.

##### 3.2.4.2 Secondary outcomes

We will assess the 5-year survival, median survival, recurrence-free survival, quality of life, and adverse events or complications of patients with resectable early NSCLC who were treated with adjuvant therapy.

### 3.3 Information sources

We will search PubMed (Medline), Embase, Google Scholar, Cancerlit, and the Cochrane Central Register of Controlled Trials for related studies published before June 20, 2019 without language restrictions.

### 3.4 Search strategy

We will use the relevant keywords or subject terms adhered to Medical Subject Heading (MeSH) terms to search for eligible studies in the electronic databases which were mentioned above without language restrictions. The PubMed search strategies are shown in Table 1.

**Table 1:**
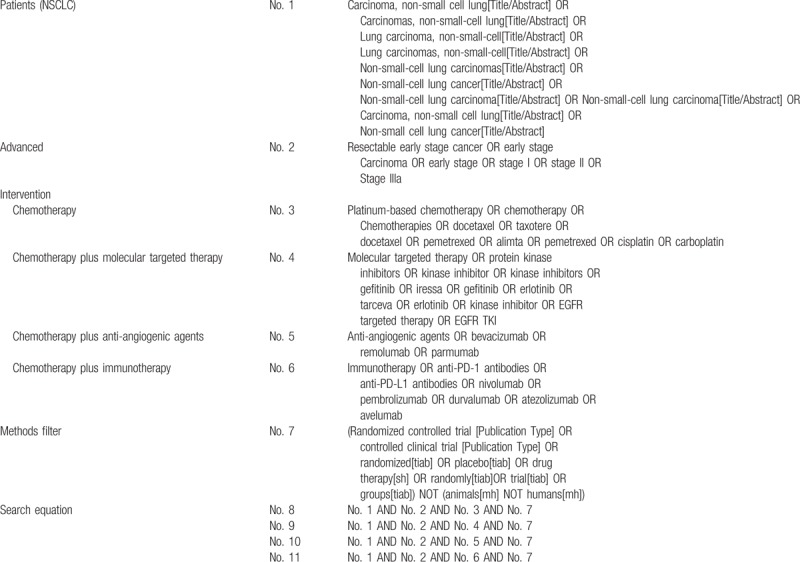
PubMed search strategies.

### 3.5 Data collection and analysis

We will utilize the measures described in the Cochrane Handbook for Systematic Reviews of Interventions to pool the evidence.^[14]^

#### 3.5.1 Study selection

Two reviewers (TCC, PPZ) will investigate each title and abstract of all literatures searched independently and identify whether the trials meet the inclusion criteria as designed and described in this protocol. Two authors (TCC, PPZ) will in duplicate and independently screen the full text of all potential eligible studies to exclude irrelevant studies or determine eligibility. The 2 reviewers will list all the studies included and document the primary reasons of exclusion for studies that do not conform to the inclusion criteria. Disagreements between the 2 authors will be resolved by discussing with the third author (JBL), if necessary, consulting with the fourth author (MQK). We will show the selection process in details in the PRISMA flow chart.

#### 3.5.2 Data extraction and management

The 2 authors (TCC, PPZ) will extract the following data independently from the studies included.

- Study characteristics and methodology: publication date, the first author, country, randomization, study design, periods of data collection, follow-up duration, total duration of study, and withdrawals, etc
- Participant characteristics: sex, age, tumor stage, pathology diagnosis, ethnicity, performance status, history of smoking, pathologic tumor size, and inclusion criteria, etc
- Interventions: type of operation, extent of resection, therapeutic means, drugs, dosage, modality and frequency of administration, etc
- Outcome and other data: overall survival, 5-year survival, median survival, disease-free survival, 95% confidence intervals, recurrence time, quality of life, adverse events, and complications, etc

We will record all the date extracted in a pre-designed table and consult the first author of the trial by e-mail before determining eligibility, if the reported data of which are unclear or missing.

### 3.6 Assessment of risk of bias in included studies

Two authors (TCC, PPZ) will use the Cochrane Handbook for Systematic Reviews of Interventions to assess the risk of bias of each study included independently based on the following ranges: random sequence generation (selection bias); allocation concealment (selection bias); blinding of participants and personnel (performance bias); blinding of outcome assessment (detection bias); incomplete outcome data (attrition bias); selective outcome reporting (reporting bias); other bias.^[13]^ Each domain will be assessed as high, low, or uncertain risk of bias. The results and details of assessment will be reported on the risk of bias graph.

### 3.7 Data analysis

The data will be synthesized by Review Manager 5.3 software. We will conduct a systematic review and meta-analysis only if the data gathered from included trials are judged to be similar enough to ensure a result that is meaningful. The Chi-square test and *I*^2^ statistic will be used to assess statistical heterogeneity among the trials included in matched pairs comparison for standard meta-analysis. The random effect model will be applied to analyze the data, if there is substantial heterogeneity (*P* < .1 or *I*^2^ statistic >50%) and the trials will be regarded to be obvious heterogeneous. Otherwise, we will utilize fixed effect model to analyze the data. Mantel-Haenszel method will be adopted to pool of the binary data. The results will be reported in the form of relative risk (RR) between 95% confidence interval (CI) of the date. The continuous data will be pooled by inverse variance analysis method and the results will be shown in the form of standardized mean difference (SMD) with 95% confidence interval (CI) of the date.

#### 3.7.1 Subgroup analysis

If there is high heterogeneity (*I*^2^ statistic > 50%) and the data are sufficient, subgroup analysis will be conducted to search potential causes of heterogeneity. Subgroup analysis will be performed in different methods of postoperative adjuvant therapy, ethnicity, history of smoking, tumor stage, and type of operation.

#### 3.7.2 Sensitivity analysis

Sensitivity analysis will be conducted to assess the reliability and robustness of the aggregation results via eliminating trials with high bias risk.

### 3.8 Publication bias

If there are ≥10 trials included, we will construct a funnel plot and use Egger test to assess publication bias. If reporting bias is suspected, we will consult the study author to get more information. If publication bias does exist, we will apply the fill and trim method to analyze publication bias in the trials.^[15]^

### 3.9 Evidence evaluation

We will evaluate all the evidence according to the criteria of GRADE (imprecision, study limitations, publication bias, consistency of effect, and indirectness bias). The quality of all evidence will be evaluated as 4 levels (high, moderate, low, and very low).^[16]^

## 4 Discussion

The 5-year survival rate of patients with pathological stage Ia to IIIa NSCLC after surgery is 73% to 25% and there is no appropriate cure for postoperative recurrence or metastasis of NSCLC. Postoperative adjuvant therapy for patients with early resectable NSCLC is necessary to improve the overall survival rate and quality of life of patients. With the emergence of molecular targeted therapy and immunotherapy, the postoperative adjuvant therapy has become diversified. The integration of different treatment modalities for early NSCLC patients remains a challenge for clinical oncologists. Therefore, a thorough discussion of the diversity of adjuvant therapies is needed to determine the optimal treatment strategy for each patient with resectable NSCLC. The purpose of this study is to provide reliable evidence for integration of different treatment modalities and making the optimal treatment strategies for patients with early NSCLC.

## Author contributions

**Conceptualization:** Tianci Chai, Peipei Zhang.

**Data curation:** Tianci Chai, Peipei Zhang, Zhenyang Zhang.

**Formal analysis:** Tianci Chai, Peipei Zhang, Zhenyang Zhang.

**Funding acquisition:** Mingqiang Kang, Jiangbo Lin.

**Investigation:** Tianci Chai, Peipei Zhang.

**Methodology:** Tianci Chai, Peipei Zhang, Zhenyang Zhang.

**Project administration:** Peipei Zhang.

**Resources:** Tianci Chai, Peipei Zhang, Yuhan Lin, Wenwei Lin.

**Software:** Tianci Chai, Peipei Zhang, Yuhan Lin.

**Supervision:** Mingqiang Kang, Jiangbo Lin.

**Validation:** Tianci Chai, Yuhan Lin, Wenwei Lin.

**Visualization:** Tianci Chai, Wenwei Lin.

**Writing – original draft:** Tianci Chai.

**Writing – review & editing:** Tianci Chai, Wenwei Lin, Mingqiang Kang, Jiangbo Lin.
